# Effects of Zinc Supplementation on Inflammatory and Cognitive Parameters in Middle-Aged Women with Overweight or Obesity

**DOI:** 10.3390/nu15204396

**Published:** 2023-10-17

**Authors:** Liziane da Silva de Vargas, Jeferson Jantsch, Juliana Ribeiro Fontoura, Gilson Pires Dorneles, Alessandra Peres, Renata Padilha Guedes

**Affiliations:** 1 Programa de Pós-Graduação em Biociências, Universidade Federal de Ciências da Saúde de Porto Alegre (UFCSPA), Rua Sarmento Leite, 245, Porto Alegre 90050-170, Brazil; lizianev@ufcspa.edu.br (L.d.S.d.V.); jefersonj@ufcspa.edu.br (J.J.); peres@ufcspa.edu.br (A.P.); 2 Graduação em Nutrição, Universidade Federal de Ciências da Saúde de Porto Alegre (UFCSPA), Rua Sarmento Leite, 245, Porto Alegre 90050-170, Brazil; 3 Hospital Moinhos de Vento, Rua Ramiro Barcelos, 910, Porto Alegre 90035-000, Brazil; gilson.dorneles@gmail.com; 4 Programa de Pós-Graduação em Ciências da Saúde, Universidade Federal de Ciências da Saúde de Porto Alegre (UFCSPA), Rua Sarmento Leite, 245, Porto Alegre 90050-170, Brazil

**Keywords:** zinc supplementation, cognitive impairment, obesity

## Abstract

Obesity has been linked to cognitive decline and adverse effects on brain health. Zinc (Zn) is a mineral with important metabolic functions that can modulate obesity-related neurological impairment. Thus, the present study aimed to evaluate the effects of 12 weeks of Zn supplementation on the inflammatory profile, cognitive function, and mood of overweight or obese women through a double-blind, placebo-controlled study. The study included 42 women aged between 40 and 60, randomly divided into two groups: Zn supplementation (30 mg/day) or placebo for 12 weeks. Data regarding sociodemographic, anthropometric, dietary, and physical activity were collected. Mini-mental state examination (MMSE), verbal fluency test, clock drawing test, and Stroop test were performed. Anxiety and depression symptoms were assessed using the Beck anxiety inventory and the BDI-II, respectively. Saliva samples were collected to evaluate IL-1β, IL-6, TNF-α, insulin, nitrite, and Zn levels. Of the 42 participants (mean age 49.58 ± 6.46 years), 32 were included in the study analyses. Changes in body weight and macronutrient consumption were not different between placebo and Zn supplementation groups. Cognitive scores on the MMSE and Stroop tests were higher in the Zn supplementation group than in the placebo group. Salivary levels of IL-1b and Zn increased in the Zn group compared to placebo. There was no significant change in the adjusted means of the BDI-II and BECK scores between the zinc vs. placebo groups. Twelve weeks of Zn supplementation was able to partially improve the cognitive scores assessed in overweight or obese women, regardless of weight loss. These findings suggest that Zn supplementation can be considered an adjunct strategy to enhance cognitive health in overweight or obese women.

## 1. Introduction

Zinc (Zn) plays an essential role in biological processes, including modulation of gene transcription, cell signaling pathways, enzyme activity, and regulation of metabolic and brain function [1]. Its role in insulin synthesis, storage, and secretion is well known, as is its involvement in insulin sensitivity [2]. In the brain, Zn participates in synaptic plasticity through intracellular and intercellular processes and is also released into the synaptic cleft as a neurotransmitter [3]. Considering the importance of Zn in a wide variety of physiological processes, its homeostasis in the body is extremely important. Zinc is regulated by complex mechanisms of absorption, transport, and excretion, which aim to keep zinc levels in balance. Deregulations in this homeostasis can lead to deficiencies or excesses of zinc concentrations, both harmful to the central nervous system (CNS) and health [4,5].

It has been stated that zinc deficiency is associated with comorbidities such as obesity, metabolic and cardiovascular diseases, and cognitive decline [6,7,8,9]. Studies have shown that people with obesity have lower serum Zn levels [10,11]. Furthermore, Zn supplementation was associated with a significant reduction in body weight in overweight or obese individuals [12,13]. These effects can be attributed to zinc’s ability to decrease inflammation and oxidative stress and its role in modulating lipid and glucose metabolism [14]. Furthermore, zinc plays a crucial role in regulating hormonal function, including testosterone production, which in turn influences lipid metabolism and body composition [15].

Zinc deficiency in obesity may further contribute to a chronic inflammatory state and increase the susceptibility to related complications [16]. The pro-inflammatory milieu affects the entire body, including the CNS [17], worsening cognitive and executive functions. It is worth mentioning that obesity is a risk factor for cognitive decline [18].

Recently, the relationship between increased body mass index (BMI) and waist circumference (WC) with cognitive decline and dementia in the elderly was described [19,20]. In addition, Zn supplementation was able to reverse short-term memory deficits in rats [21] and increase antioxidant and anti-inflammatory capacity in both animals and humans [21,22]. However, the effects of Zn on cognition in clinical studies are still unclear.

Considering that Zn supplementation may minimize metabolic dysfunction and thus exert neuroprotective effects, reducing obesity-related cognitive impairment, the present study aimed to evaluate the impact of zinc supplementation on inflammatory and cognitive parameters in middle-aged overweight or obese women aged 40 to 60 years.

## 2. Materials and Methods

The present study was a pilot, randomized, double-blind, placebo-controlled study conducted in Porto Alegre, RS, Brazil. The study was approved by the ethics and research committee of the Federal University of Health Sciences of Porto Alegre (UFCSPA), under approval number 4.031.047. The study follows the ethical precepts established by Resolution 196/96 of the National Health Council, which regulates ethics in research with human beings. The study protocol was designed and conducted in accordance with the Declaration of Helsinki, and all participants gave informed consent before participating in the study.

The sample size calculation was determined using the Randomized Controlled Trials formula proposed by Chan (2003), as described in [23]. The calculated sample size was 21 per group after considering a 20% dropout rate.

### 2.1. Participants and Randomization

The eligibility criteria in this study were as follows: overweight or obese women (BMI ≥ 25 kg/m^2^), aged between 40 and 60 years, with 6 years or more of formal education, and without significant weight change in the 6 months before inclusion in the study. Participants were recruited through local advertisements and social media.

**Figure 1:**
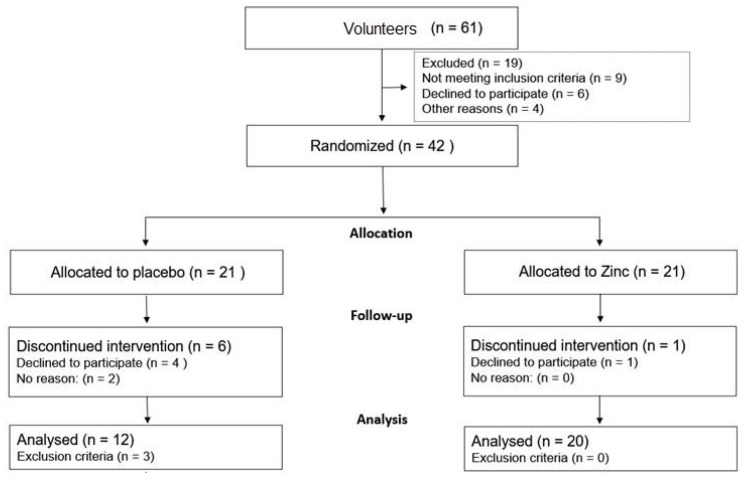
Flowchart of study participants.

Sixty-one people enrolled to participate in the study. After eligibility assessment, 42 volunteers were randomly allocated to receive placebo/control capsules (*n* = 21) or zinc capsules/intervention (*n* = 21) for 12 weeks, as shown in Figure 1. To be eligible to participate, people should not be elitist (alcohol dependent), not suffer from chronic diseases (diabetes mellitus, neurological, kidney, liver, or gastrointestinal disorders), not be undergoing hormone replacement, not have a history of stroke, nor be pregnant. In addition, participants must not have taken antibiotics, anti-inflammatories, probiotics, zinc, or omega-3 supplements, multivitamins, or multimineral at least one month before starting the study. The study did not include confirmed cases of COVID-19 or those with any usual symptoms of COVID-19. Exclusion criteria were as follows: any change in the regular diet, medication, and physical activity, occurrence of side effects that would cause interruption of the intervention, and non-adherence to the research instructions.

Figure 1 presents the study flowchart. Over the 12-week study period, there were 9 dropouts in the placebo group and 1 in the Zn supplement group; thus, the sample size at the end was 12 people in the placebo group and 20 in the Zn group.

### 2.2. Participant Interview and Sample Collection Procedures

Initially, volunteers completed an online pre-registration form with a study description. Each volunteer was contacted by phone to explain the objectives and criteria for participating in the study. After the eligibility analysis, the volunteers were randomly allocated, according to the order of registration, to Group A (odd numbers) or Group B (even numbers). The survey was carried out during the COVID-19 pandemic; thus, social distancing was necessary during data collection. For this reason, a kit was delivered to the participant’s residence containing the following: the informed consent form, a salivary collection kit with a guideline for use, 3-day food log, self-reported inventories (BECK scale, BDI-II), a blank sheet, and a bottle containing 30 placebo or Zn capsules for the first 30 days of the study.

After the kit delivery, an online meeting was conducted for anamnesis, application of cognitive tests, and to give instructions about the acquisition of anthropometric measurements, completion of the inventories, and saliva collection.

At the end of the first month of the study, delivery of placebo or Zn additional capsules was scheduled (2 bottles, 1 containing 30 capsules and the other containing 32 capsules). After 12 weeks of intervention, all tests were repeated (final evaluation). During the intervention, participants were instructed to maintain a constant diet, physical activity, and medication. No weight loss or specific diets were provided during the intervention period. At the end of the study, all participants received individualized nutritional guidance.

Data were collected before and after 12 weeks of intervention. At the end of the intervention, a final visit was scheduled to obtain post-intervention saliva samples and the material with the assessments self-completed by the participants.

### 2.3. Salivary Sample Collection and Analysis

All participants were instructed to collect the salivary sample after an overnight fast, between 8 a.m. and 10 a.m., two hours after brushing their teeth. Saliva was collected using the passive drool method (unstimulated saliva). This protocol is the most recommended method, as most analytes can be easily quantified by traditional quantification methods [24]. All samples were stored at −20 °C until further processing.

The levels of cytokines IL-1, IL-6, TNF-α, MCP-1, and insulin were evaluated via enzyme-linked immunosorbent assay using a commercially available MILLIPLEX^®^ kit. The assay was performed according to the manufacturer’s instructions. The nitrite dosage was performed according to the protocol described by Miranda et al. (2001) [25]. Zn content was determined via flame atomic absorption spectrometry, as previously described in [26]. To perform the analysis, the analytical curve was performed with a standard 1000 mg/L Zn stock solution (Merck, Kenilworth, NJ, USA), with a purity of 99.9%.

### 2.4. Zn Supplement

Zinc and placebo capsules were manufactured by Plenna Pharmacy, Porto Alegre, Brazil. Each zinc supplement capsule contained 30 mg of chelated zinc (zinc bisglycinate 20%; excipients: magnesium stearate, talc, aerosil, sodium lauryl sulfate, and starch), while each placebo capsule contained 30 mg of cornstarch. Both were similar in size, shape, color, weight, and packaging. Bottles containing 30 capsules were sealed and identified by the pharmacy as Group A or Group B. Therefore, neither the participants nor the researchers knew how to distinguish which group was receiving Zn or placebo. At the end of the study, the description of the groups (intervention or placebo) was revealed to the researchers.

Participants were advised to take one capsule daily, 60 min after a main meal, for 12 weeks. In the first 20 days, reminders via phone messages were sent daily to the participants. Participants were asked to record any adverse events while taking the supplement and were in contact with a researcher daily.

### 2.5. Cognitive Assessment

Cognitive function was assessed using a battery of neurocognitive tests with established reliability and validity. The battery included mini-mental state examination (MMSE) [27], clock drawing test [28], verbal fluency test (TFV) animal category [29], Stroop test [30], Beck depression inventory (BDI-II) [31], and Beck anxiety inventory (BAI) [32]. All tests were performed through an online meeting [33,34], administered and scored by a trained researcher, with an approximate duration of 1 h and 30 min. Based on cognitive tests, the following domains were examined: spatial and temporal orientation, immediate memory, executive functions, special visual abilities, recall, attention, and language.

### 2.6. Statistical Analysis

Data were analyzed using SPSS software. Data normality was analyzed using the Shapiro–Wilk test. Mean ± SD and median (IC 95%) were used to present normally and non-normally distributed variables, respectively. Mean differences from baseline between the supplement and the placebo group were analyzed using the independent t test for continuous parameters. Interaction effects were analyzed using a two-way repeated measure analysis of covariance (ANCOVA) adjusted for baseline with Bonferroni correction.

In addition, the mean intra-group percentage change was calculated using the formula [(mean at week 12 − mean baseline)/mean baseline × 100%] and presented in line graphs. The Wilcoxon test was applied, and the *p*-value corrected using Bonferroni.

## 3. Results

The baseline characteristics of the study participants are summarized in Table 1. A total of 32 female participants, with a mean age of 49.58 ± 6.46 years, were randomly allocated into two groups: a placebo group consisting of 12 participants with a mean age of 51.06 ± 6.90 years and a Zn supplement group consisting of 20 participants with a mean age of 48.40 ± 6 years. The majority of participants had completed at least twelve years of formal education, accounting for 59.4% of the total sample.

The average body weight of the participants was 89.5 ± 16.1 kg, and the mean BMI was within the obesity range at 34.0 ± 6.34 kg/m^2^. A minority of participants, representing only 31.2%, reported engaging in physical activity at least twice a week. The average duration of sleep among all participants was 7 ± 1.5 h. Baseline values for body weight, BMI, cognitive test scores, biomarkers analyzed, and dietary habits did not exhibit significant differences between the two groups, suggesting a well-executed randomization process.

Table 2 presents the results of the intervention, with comparisons made between the placebo and Zn groups after adjusting for baseline data. According to the ANCOVA analysis, Zn supplementation did not yield statistically significant effects on body weight (*p* = 0.807), BMI (*p* = 0.958), calorie intake (*p* = 0.265), macronutrient consumption (including carbohydrates, proteins, and lipids) (*p* > 0.05), or dietary zinc intake (*p* = 0.222) when compared to the placebo group.

**Figure 2:**
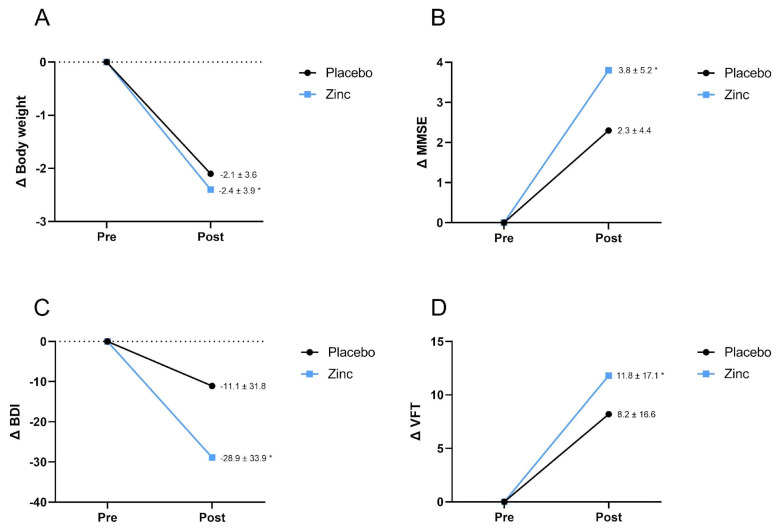
Comparison of pre- and post-intervention percentage change within the same group. (**A**) Body weight variation, (**B**) MMSE score, (**C**) BDI score variation, (**D**) VFT score variation. * Significant at *p* < 0.05 using Wilcoxon test.

However, while no significant differences were observed between the Zn supplement and placebo groups in terms of intervention effects, the intragroup Wilcoxon test showed a significant reduction in body weight within the Zn group (−2.4 ± 3.9, *p* = 0.019). Conversely, in the placebo group, there was no notable change in body weight (2.1 ± 3.6, *p* > 0.05) (see Figure 2A).

Table 3 illustrates the impact of Zn supplementation on cognition and emotional status. Notably, Zn supplementation had a significant effect on MMSE and Stroop test scores. Specifically, the mean MMSE score was significantly higher in the Zn-supplemented group (28.46 (95% CI 27.95–28.97)) in comparison to the placebo group (27.57 (95% CI 26.91–28.94)), indicating improved performance on the test following supplementation. Regarding the Stroop test, the score in the Zn group was 61.97 (95% CI 59.08–64.86), while in the placebo group, it was 68.13 (95% CI 64.39–71.87), suggesting an enhancement in cognitive performance after Zn supplementation. Furthermore, intragroup analyses were conducted to assess mean differences between post-test and pre-test scores. In the Zn group, there was a significant improvement in MMSE test scores (3.8 ± 5.2, *p* = 0.021), BDI (−28.9 ± 33.9, *p* = 0.004), and TFV (11.8 ± 17.1, *p* = 0.036) when compared to baseline values, indicating enhanced cognitive performance. In contrast, the placebo group did not exhibit such improvements (*p* > 0.05). These data are presented in line graphs (Figure 2B–D).

Table 4 presents the impact of Zn supplementation on the biomarkers analyzed in the saliva samples. An analysis of covariance (ANCOVA) revealed noteworthy findings: there were significant differences in the levels of IL-1b (*p* = 0.013) and Zn (*p* = 0.038) between the two groups following the 12-week intervention. Conversely, the remaining analytes, namely IL-6, insulin, MCP-1, TNFα, and nitrite, did not exhibit significant differences when comparing the Zn and placebo groups.

## 4. Discussion

Our study demonstrated that a 12-week period of zinc supplementation in overweight or obese adult women led to improved cognitive performance, as indicated by the results obtained from the MMSE and Stroop test. However, this intervention did not produce any significant changes in body weight.

Our findings of cognitive improvement are corroborated by previous studies that have shown neuroprotective properties, anti-inflammatory, and antioxidant effects of Zn [35,36]. The imbalance in zinc ion (Zn^2+^) levels appears to play a crucial role in the etiology and progression of neurodegenerative diseases [5], such as Alzheimer’s disease (AD), Parkinson’s, attention deficit hyperactivity disorder (ADHD) [37,38,39,40], as well as psychiatric disorders [41]. Zinc deficiency is associated with AD, while Zn supplementation has been linked to a reduced prevalence of AD and slowed disease progression [37]. Experimental studies in rodents have shown beneficial effects of Zn supplementation on physiological aspects related to the central nervous system (CNS) [42].

The protective effect exerted by zinc could be related to its role in various neurobiological processes, including the modulation of neurogenesis, neuronal migration, differentiation, and synaptic transmission, which are directly related to cognitive health [43]. It has been demonstrated that even low doses of Zn, supplemented for 4 weeks, can reduce neuroinflammation and memory deficits resulting from diet-induced obesity [26]. In line with these findings, Hafez et al. (2023) showed that Zn supplementation at both low and high doses, for the same 4-week period, was able to reverse the reduction in brain-derived neurotrophic factor (BDNF) levels in the hippocampus of obese mice [44]. In addition, a positive correlation between serum levels of BDNF and zinc has been previously described [42].

Consistently, a clinical intervention study spanning 12 weeks and involving overweight or obese subjects demonstrated a significant increase in serum BDNF levels in the group receiving Zn supplementation compared to the placebo group. Additionally, the Beck depression inventory (BDI) score exhibited a significant reduction in the number of participants receiving zinc supplementation. Interestingly, this same study unveiled an inverse correlation between BDNF levels and the severity of depression across all participants [45]. In our study, we also observed a significant decrease in BDI scores within the intervention group following Zn supplementation. However, in the comparative analysis between groups, we did not detect statistically significant results. This lack of significance could be attributed to sample losses or the fact that we utilized the overall score rather than classifying the degree of depression. Previous findings have indicated that BDI scores decreased solely in the subgroup of subjects exhibiting depressive symptoms, but not among non-depressed participants [45]. Notably, this body of evidence suggests that zinc supplementation may offer benefits in addressing the cognitive and mood impairments associated with obesity.

Obesity is a prevalent and recurring pathological condition, with alarming prevalence rates worldwide, affecting individuals across the age spectrum, encompassing children, adolescents, and adults [46]. In addition to the well-documented metabolic risks, obesity has been consistently linked to cognitive impairment [47,48,49], regardless of age [50]. However, the precise mechanisms underpinning this association remain incompletely elucidated. Current knowledge indicates that the inflammation associated with obesity exerts effects on the central nervous system, notably impacting regions such as the hippocampus, cerebral cortex, and amygdala [51,52]. These brain regions are particularly susceptible to the detrimental consequences of inflammation, which can lead to adverse effects on cognitive function [53]. In a longitudinal study encompassing 6401 middle-aged adults aged between 39 and 63 years, cognitive decline, as measured by the global cognition score, was notably more pronounced among individuals classified as obese, in comparison to their normal-weight counterparts [54]. Conversely, when investigating the impact of weight loss on global cognition in obese adults participating in a comprehensive multidisciplinary weight reduction program, it was observed that the magnitude of weight loss did not exhibit a significant correlation with cognitive improvement [55]. These findings are in concordance with our own study results, which suggest that the potential cognitive benefits attributed to Zn supplementation are independent of weight loss.

Moreover, in the present study, while Zn supplementation did not yield a statistically significant effect on weight loss when comparing the Zn and placebo groups, an intragroup analysis showed a noteworthy reduction in weight within the Zn supplementation group itself. The lack of significance in the intergroup analysis could be attributed to several factors, including the relatively small sample size in the placebo group and the duration of the intervention, which was shorter in comparison to the timelines documented in other studies [56]. However, consistent with our results, a recent systematic review and meta-analysis demonstrated that Zn supplementation was not associated with a significant effect on weight loss [13].

Although, to the best of our knowledge, there are no clinical studies evaluating the effect of zinc supplementation on inflammatory and cognitive parameters in overweight or obese women, a previous study demonstrated that zinc supplementation combined with a calorie-restricted diet was able to reduce body weight, inflammatory markers, and insulin resistance in obese individuals after a 15-week intervention. This finding suggests that Zn supplementation combined with dietary modifications may hold promise in the context of obesity therapy [56]. The results regarding Zn supplementation on body weight appear to be controversial, largely due to the heterogeneity among study populations, administered doses, intervention duration, and the presence of concomitant comorbidities. These factors collectively contribute to the challenge of making direct comparisons and arriving at definitive conclusions based on individual studies. Furthermore, it is important to note that previous studies have indicated a positive correlation between serum zinc levels and total testosterone, with evidence suggesting that moderate Zn supplementation plays a significant role in increasing androgens, which may influence body weight in males [57].

We observed a slight yet significant increase in IL-1β levels in the Zn supplementation group. Previous research has indicated that Zn has a substantial impact on inflammasome regulation and IL-1β production in both innate and adaptive immune cells [58]. Zinc deficiency has been associated with elevated proinflammatory cytokine levels, particularly IL-1β, through the activation of inflammasome signaling pathways [59]. Driessen et al. [60] also demonstrated enhanced production of IL-1β in mononuclear cells incubated with Zn, highlighting the promising immunomodulatory action of this micronutrient on the IL-1β pathway. In elderly individuals, Zn supplementation has been associated with a non-significant increase in IL-1β levels alongside a lower incidence of opportunistic infections [58]. These findings collectively suggest that Zn supplementation may possess immunometabolic properties that enhance IL-1β levels through the metabolic reprogramming of monocytes in adults. It is important to note that temporary increases in IL-1β do not necessarily indicate a negative effect of Zn supplementation. The body’s inflammatory response can be complex and multifaceted, and the long-term effects of Zn supplementation in obesity should be considered in conjunction with other health markers and the overall study context.

On the other hand, preclinical studies conducted in animal models have consistently yielded evidence regarding the advantages of Zn supplementation in ameliorating metabolic outcomes associated with obesity [26,44,61]. Obese rats supplemented with Zn showed an improvement in blood glucose levels, triglycerides, and a reduction in leptin resistance when compared to controls that did not receive Zn treatment [44]. In our study, we did not find an improvement in the analyzed inflammatory parameters. Given the limitations of blood collection due to the social distancing imposed by the COVID-19 pandemic, we instructed the participants to collect their saliva since it is a widely used alternative biological matrix. However, it is important to mention that despite the detailed information about the sample collection protocol, failures may occur that could interfere with the results. Nevertheless, saliva is an important biological fluid that has recently been used to identify biomarkers that signal early cognitive impairment in individuals with Alzheimer’s disease and potentially other neurodegenerative disorders [62,63]. In addition, the observed discrepancy in MCP-1 levels between the Zn and placebo groups before the intervention underscores another limitation of our study, suggesting the presence of some inflammatory processes in certain individuals within the Zn group. This observation is important and should be considered in the interpretation of our results. However, it is worth noting that the levels of other cytokines were similar between the groups, and evaluating inflammation based solely on a single cytokine (MCP-1) may lead to misinterpretations. Thus, our study considered other markers and parameters to comprehensively assess the effects of Zn supplementation.

## 5. Conclusions

In summary, obesity exerts detrimental effects on the central nervous system, mediated by inflammatory processes and metabolic and hormonal dysregulations. It is imperative to underscore that the prevention and management of obesity are pivotal for ameliorating its deleterious impact on cerebral functions. Therefore, the study of Zn supplementation in the context of obesity is highly relevant, given its global prevalence. The findings of the present study indicate that zinc supplementation may confer a beneficial influence on cognition among overweight women, independently of any weight loss intervention. However, it is important to emphasize that the sample size of the study was limited, which may influence the generalizability of the results. Therefore, future studies with a larger sample size are needed to provide more evidence on the effects of zinc on body weight. Despite this, the results obtained so far support the idea that zinc can be a potential supplement to protect against cognitive impairment associated with obesity.

## Figures and Tables

**Table 1:** Baseline characteristics of study participants.

	Placebo Group	Zinc Group	*p*-Value
Age ^a^	51.1 ± 6.9	48.4 ± 6.0	0.224
≥12 years of education ^b^ *n* (%)	6 (37.5)	13 (65.0)	0.101
Body weight (kg) ^b^	87.56 ± 15.26	91.08 ± 15.45	0.499
Height ^a^	1.62 ± 0.07	1.63 ± 0.05	0.616
BMI (kg/m^2^) ^b^	33.70 ± 5.58	34.51 ± 6.56	0.698
Physical activity (2 times/week) ^b^ *n* (%)	2 (12.5)	8 (40.0)	0.133
Hours of sleep ^a^	7.0 ± 1.0	6.8 ± 1.8	0.690
Scores obtained in the Cognitive tests			
MMSE ^b^	26.81 ± 1.11	27.60 ± 1.47	0.084
TFV ^a^	18.94 ± 5.35	20.65 ± 4.76	0.317
Clock test ^b^	8.60 ± 1.92	9.10 ± 1.21	0.352
Stroop test ^b^	78.19 ± 10.77	77.85 ± 21.09	0.954
BDI-II ^b^	12.55 ± 7.09	9.11 ± 6.34	0.187
BECK ^a^	16.64 ± 13.73	11.53 ± 7.86	0.226
Biomarkers ^b^			
Zinc (µg/dL)	50.8 ± 9.8	49.5 ± 7.7	0.703
Nitrite (μMol/L)	9.5 ± 3.1	10.8 ± 4.2	0.190
IL-1β (pg/mL)	187.1 ± 162.7	150.3 ± 117.2	0.922
IL-6 (pg/mL)	18.4 ± 5.8	20.1 ± 6.3	0.337
Insulin (pg/mL)	387.4 ± 575.6	406.0 ± 344.7	0.076
MCP-1 (pg/mL)	519.8 ± 1061.2	2327.7 ± 1806.8	0.009 *
TNFα (pg/mL)	3.20 ± 1.90	4.10 ± 3.7	0.909
Food record ^b^			
Energy (kcal)	1365.9 ± 323.6	1452.4 ± 417.4	0.547
Carbohydrate (kcal)	625.4 ± 201.7	675.4 ± 225.9	0.537
Protein (kcal)	280.3 ± 46.9	299.8 ± 79.5	0.450
Fat (kcal)	457.9 ± 144.2	455.5 ± 196.9	0.971
Zinc (mg)	7.12 ± 1.83	7.01 ± 2.3	0.0893

Data are presented as mean ± standard deviation or *n* (%). Continuous variables are expressed as mean ± standard deviation. Categorical variables (educational level and physical activity) are expressed as number and (%). ^a^ Student’s unpaired *t*-test for continuous variables and the ^b^ Mann–Whitney test were used to compare baseline characteristics between placebo and Zn groups. * *p* < 0.05.

**Table 2:** Effect of Zn supplementation on body weight and food intake after 12 weeks of intervention.

	Group	Adjusted Mean	CI 95%	Δ	CI 95% Δ	*p*-Value
Weight (kg)	Placebo	87.69	(85.72–89.65)	−0.300	(−2.79–2.19)	0.807
	Zinc	87.39	(85.87–88.90)			
BMI (kg/m^2^)	Placebo	33.26	(32.47–34.05)	−0.026	(−1.02–0.97)	0.958
	Zinc	33.23	(32.62–33.84)			
Energy (kcal)	Placebo	1245.70	(1036.95–1454.44)	145.34	(−118.1–408.7)	0.265
	Zinc	1391.03	(1235.64–1546.43)			
Carbohydrate (kcal)	Placebo	547.67	(457.49–637.84)	75.75	(−38.40–189.90)	0.183
	Zinc	623.41	(556.46–690.37)			
Protein (kcal)	Placebo	270.02	(215.41–324.63)	22.46	(−46.08–91.00)	0.504
	Zinc	292.48	(251.65–333.31)			
Fat (kcal)	Placebo	378.60	(280.83–476.37)	48.66	(−73.63–170.96)	0.418
	Zinc	427.26	(353.97–500.55)			
Zinc (mg)	Placebo	6.47	(4.98–7.97)	1.13	(−0.736–3.01)	0.222
	Zinc	7.61	(6.49–8.73)			

Δ: Comparison between groups after the treatment period with correction for baseline values using ANCOVA. CI 95%.

**Table 3:** Effect of Zn supplementation on mental status after 12 weeks of intervention.

	Group	Adjusted Mean	CI 95%	Δ	CI 95% Δ	*p*-Value
MMSE	Placebo	27.57	(26.91–28.24)	0.885 *	(0.03–1.74)	0.044 *
	Zinc	28.46	(27.95–28.97)			
VFT	Placebo	21.23	(19.23–23.24)	0.877	(−1.69–3.44)	0.490
	Zinc	22.11	(20.57–23.65)			
Clock Test	Placebo	9.20	(8.76–9.64)	0.283	(−0.27–0.84)	0.306
	Zinc	9.48	(9.14–9.82)			
Stroop test ^a^	Placebo	68.13	(64.39–71.87)	−6.156	(−10.90–−1.42)	0.013 *
	Zinc	61.97	(59.08–64.86)			
BDI-II ^a^	Placebo	12.86	(9.85–15.88)	2.276	(1.89–0.24)	0.239
	Zinc	15.14	(12.80–17.48)			
Beck scale ^a^	Placebo	12.59	(7.08–18.10)	−2.340	(−9.37–4.69)	0.500
	Zinc	10.25	(5.95–14.55)			

Data corrected for baseline. Analysis of covariance (ANCOVA) used to test post-test differences between groups. Δ Difference between the post-test between the groups. CI 95% *p* < 0.05 considered significant. Mini-mental state examination (MMSE). Verbal fluency test (VFT). Beck depression inventory (BDI-II). ^a^ A higher score indicates a worse performance. * *p* < 0.05.

**Table 4:** Effect of zinc supplementation salivary biomarkers after 12 weeks of intervention.

	Group	Adjusted Mean	CI 95%	Δ	CI 95% Δ	*p*-Value
IL-1β (pg/mL)	Placebo	84.38	(20.76–148.01)	118.234 *	(32.06–204.41)	0.013 *
	Zinc	202.62	(144.54–260.69)			
IL-6 (pg/mL)	Placebo	18.88	(16.32–21.43)	−2.271	(−5.53–0.99)	0.163
	Zinc	16.60	(14.58–18.62)			
Insulin (pg/mL)	Placebo	308.07	(90.40–525.73)	132.579	(−211.92–477.08)	0.393
	Zinc	440.65	(173.98–707.318)			
MCP-1 (pg/mL)	Placebo	332.23	(164.90–499.55)	−2.277	(−223.87–−219.31)	0.983
	Zinc	329.95	(199.23–460.66)			
TNFα (pg/mL)	Placebo	3.58	(1.81–5.35)	0.223	(−2.03–2.48)	0.840
	Zinc	3.80	(2.41–5.20)			
Nitrite (μMol/L)	Placebo	12.95	(8.96–16.95)	−1.078	(−6.10–−3.95)	0.663
	Zinc	11.88	(8.84–14.91)			
Zinc (µg/dL)	Placebo	35.37	(26.95–43.78)	11.678	(0.71–22.65)	0.038 *
	Zinc	47.05	(40.05–54.04)			

Adjusted mean for pre-test. Analysis of covariance (ANCOVA) used to test post-test differences between groups. Δ Difference between the post-test between the groups. * *p* < 0.05 considered significant.

## Data Availability

All data relating to the present study are available in this manuscript.
